# Variations in phenolic acid metabolites among *Forsythia suspensa* populations in response to environmental heterogeneity

**DOI:** 10.3389/fpls.2025.1683181

**Published:** 2025-11-05

**Authors:** Shanshan Zhou, Zhengsen Li, Qingyu Wang, Tianyu Cao, Danyang Li, Kailu Li, Jinlan Ji

**Affiliations:** Faculty of Environmental Science and Engineering, Shanxi Institute of Science and Technology, Jincheng, China

**Keywords:** *Forsythia suspensa*, phenolic acid, metabolic profiling, environmental variables, environmental heterogeneity

## Abstract

**Introduction:**

*Forsythia suspensa* (Thunb.) Vahl,a pharmacopoeial medicinal plant,is valued for its therapeutic efficacy in clearing heat and detoxifying, dispelling wind-heat, and promoting blood circulation to resolve stasis. Phenolic acids, ubiquitous secondary metabolites in *F. suspensa*, are critically linked to pharmacological activities and exhibit diverse biological functions.It is therefore of significant interest to investigate whether environmental changes also alter the content distribution of phenolic acids in *F. suspensa*.

**Methods:**

To elucidate the chemical diversity and ecological drivers of its bioactive compounds, we conducted phenolic acids metabolomic profiling across 10 wild populations *F. suspensa* using UPLC-MS/MS.

**Results:**

Results showedsignificant inter-population variation in all thirty phenolic acids analyzed. Specifically, Verbascoside was significantly enriched in the AZ population, showing a positive correlation with Mean Monthly Temperature Range, Temperature Seasonality, and Temperature Annual Range, but a negative correlation with Annual Precipitation, Precipitation of Driest Quarter, Precipitation of Coldest Quarter and Min Temperature of Coldest Month. 4-Coumaroylshikimate, accumulated in WML and PS, was positively correlated with Precipitation of Wettest Month, Precipitation of Wettest Quarter, and Precipitation of Warmest Quarter, while negatively correlated with Min Temperature of Coldest Month. Gallotannin, enriched in LT, was negatively correlated with Mean Monthly Temperature Range, Temperature Seasonality, Temperature Annual Range, and Precipitation Seasonality, but positively correlated with Annual Precipitation. Isosalicin, accumulated in HX, showed a positive correlation with Max Temperature of Warmest Month and negative correlation with Annual Precipitation and Elevation.

**Discussion:**

These findings demonstrated that phenolic acids accumulation in *F. suspensa* was primarilydriven by temperature heterogeneity, with precipitation as a secondary factor, whereas adaptation to elevation plays a minimal role. This study systematically investigated the divergence and environmental drivers of phenolic acids in *F. suspensa* populations, clarifying the molecular ecological mechanisms behind its adaptation toenvironmental heterogeneity and thereby offering important insights into how ecological factors shape the medicinal potential of *F. suspensa*, ultimately informing targeted breeding and optimized field management.

## 1 Introduction

*Forsythia suspensa* (Thunb.) Vahl. a plant belonging to the Oleaceae family, is a widely used bulk medicinal herb whose fruit, *Forsythiae Fructus*, has been pharmacologically documented since the *Shen Nong Ben Cao Jin*g (Dong et al., 2017). It is currently recognized in the pharmacopoeias of China, Japan, the United States, and South Korea (D, A. K. F. A, 2015; Ministry of Health, L. a. W, 2016; P, C. U. S, 2017; Commission, C. P, 2020). Harvested at distinct phenological stages, *Forsythiae Fructus* is categorized as Qingqiao and Laoqiao. The greenish fruits that start to ripen are known as Qingqiao, whereas the fully ripened yellow fruits are known as Laoqiao (Dong et al., 2017; Wang et al., 2018). Characterized by a bitter taste, subtle fragrance, and cold property, this herb acts on the lung, heart, and small intestine meridians. Its demonstrated bioactivities include heat-clearing, detoxification, nodule-dispersion, and wind-heat dispersion, with clinical applications spanning fever, inflammatory conditions, gonorrhea, and early-stage warm pathogen diseases (Wang et al., 2018). Revered as the “holy herb for ulcerative disorders”, it holds significant medicinal, ecological, and economic value.

Plant growth is influenced by environmental factors, to which plants respond through two principal strategies: adjustments in distribution and the generation of genomic adaptive variations (Davis and Shaw, 2001; Lei et al., 2015). Environmental changes are primarily reflected in climatic fluctuations over temporal scales and environmental heterogeneity across spatial scales (Yang et al., 2017). Environmental heterogeneity—spatiotemporal variation in abiotic (temperature, moisture, solar radiation, edaphic factors) and biotic conditions—critically modulates plant secondary metabolism. Abiotic stressors (drought, thermal extremes, UV-B) trigger adaptive responses that alter metabolite biosynthesis, including phenolic acid metabolites (Zhou et al., 2021a; Zhou et al., 2021b; Fang et al., 2022). Phytochemical studies have revealed over 300 constituents in *F. suspensa*, encompassing lignans, phenylglycosides, phenolic acids, and flavonoids, *etc* (Zhang et al., 2012; Dong et al., 2017; Wang et al., 2018; Liu et al., 2025). Among these, phenolic acids have garnered significant pharmacological interest due to their broad-spectrum bioactivities, which are well-documented and include antimicrobial, anticancer, anti-inflammatory, and anti-mutagenic effects (Aldred et al., 2009; Kumar and Goel, 2019; Lang et al., 2024).

The main source of medicinal materials for *F. suspensa* was derived from wild resources. *F. suspensa* in China is widely distributed across mountainous regions (altitude: 250–2200 m), primarily in Shanxi, Henan, Shaanxi, and Hebei provinces. Variations in growing environments and climates were the primary reasons for the significant differences in the content of bioactive metabolites among *F. suspensa* populations (Fu et al., 2014, 2016; Yang et al., 2017; Li et al., 2020). Given the extensive distribution and diversity of wild *F. suspensa* resources, along with the considerable variation in the content of medicinal active substances and the tendency for varietal changes during cultivation, the germplasm resources with high and stable levels of bioactive metabolites in *F. suspensa* have become the main constraints on the development of the industry and its pharmaceutical applications (Fang, 2015; He et al., 2023). Therefore, selecting varieties with high content of bioactive metabolites is one of the key issues in the *F. suspensa* cultivation industry.

Previous research on *F. suspensa* has predominantly focused on its chemical composition, pharmacological properties, and quantitative analysis (Zhang et al., 2012; Wang et al., 2018). Pharmacological investigations have revealed that *F. suspensa* exhibits notable anti-inflammatory, antimicrobial, and anticancer activities. Guo et al. suggested that these properties contribute to its traditional use in clearing heat and detoxification (Guo et al., 2015). Furthermore, Wang et al. reported that its antioxidant and anti-inflammatory characteristics hold promising therapeutic potential for cognitive impairment and Parkinson’s disease (Wang et al., 2013). From a biosynthetic perspective, integrated genomic and transcriptomic analyses by Li et al. proposed multiple biosynthetic pathways for forsythiaside and forsythiaside A, identifying 48 candidate genes involved in their synthesis (Li et al., 2023). In addition to its chemical and pharmacological profile, environmental factors have been shown to significantly influence the growth and quality of *F. suspensa*. Studies indicated that altitude and slope orientation critically affect both the yield and quality of cultivated plants (Wang, 2014; Chen et al., 2025). Fu et al. demonstrated a strong correlation between geographic distance and population genetic differentiation (Fu et al., 2016). Suo et al. identified precipitation as a key factor influencing growth (Suo et al., 2024), while Li et al. highlighted the impact of temperature on its geographical distribution (Li et al., 2020). Although previous studies have addressed environmental impacts on the growth and distribution of *F. suspensa*, the effect of such variations on the content of phenolic acid metabolites remains largely unexplored. It is therefore of significant interest to investigate whether environmental changes also alter the content distribution of phenolic acids in *F. suspensa*. To elucidate the environmental effects on phenolic acid metabolism, we performed UPLC-MS/MS-based metabolic profiling of phenolic acids across 10 populations of *F. suspensa*, integrating environmental data to identify the key factors governing their accumulation. This study could not only shed light on the molecular ecological basis for *F. suspensa* adapted to environmental heterogeneity, but also provide important insights into how ecological factors shape the medicinal potential of this species, ultimately informing targeted breeding and optimized field management.

## 2 Materials and methods

### 2.1 Plant growth sample collection

**Figure 1:**
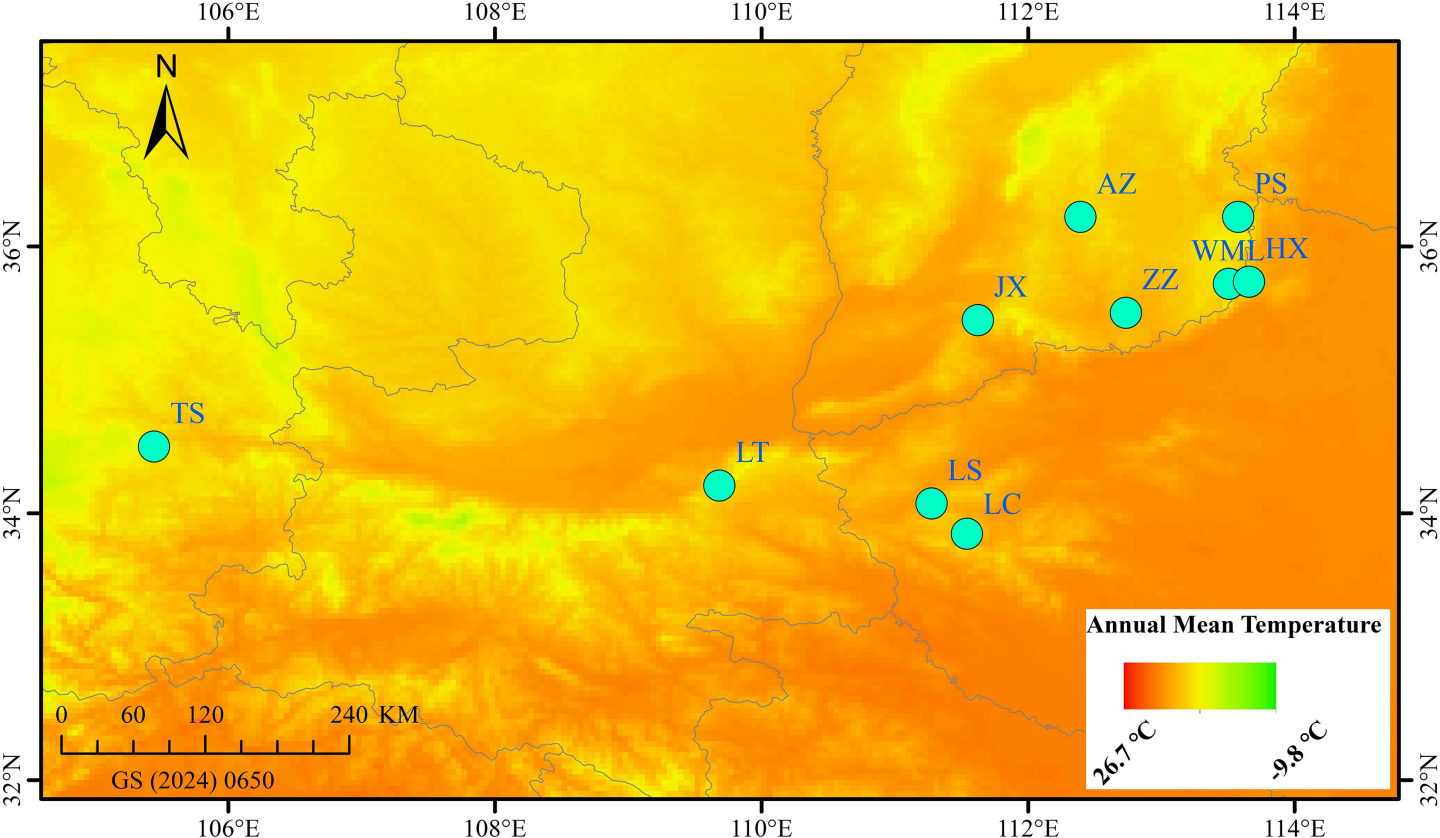
Geographical distribution of 10 *F. suspensa* populations in China.

From late July to early August 2024, fruit samples (Qingqiao) were collected from 10 wild *F. suspensa* populations spaced at least 30 km apart (**Figure 1**, **Table 1**). Three samples were randomly collected from each population, with an inter individual spacing of at least 50 m. The latitude and longitude of each population were precisely recorded using GPS (**Table 1**). The collected samples were immediately frozen in liquid nitrogen and stored at -80 °C for future use.

**Table 1:** Details of the locality information of 10 wild *F. suspensa* populations.

Code	Location	Population code	Longitude (N°)	Latitude (N°)	Elevation (m)
1	WangMangling, Shanxi	WML	113°30′20”	35°43′9”	1474
2	Zezhou, Shanxi	ZZ	112°44′3”	35°30′7”	929
3	Pingshun, Shanxi	PS	113°34′36”	36°13′18”	1349
4	Anze, Shanxi	AZ	112°23′33”	36°13′13”	1219
5	Jiangxian, Henan	JX	111°37′25”	35°26′50”	1010
6	Huixian, Henan	HX	113°39′26”	35°44′8”	781
7	Luanchuan, Henan	LC	111°32′30”	33°50′47”	1080
8	Lushi, Henan	LS	111°16′32”	34°4′21”	1016
9	Lantian, Shaanxi	LT	109°41′4”	34°12′27”	1444
10	Tianshui, Gansu	TS	105°26′35”	34°29′59”	1767

### 2.2 Phenolic acids metabolic profiling

#### 2.2.1 Preparation of sample solution

The samples of *F. suspensa* were vacuum freeze-dried for 63 hours in a freeze dryer (Scientz-100F). For each sample, 50 mg homogenized sample powder was suspended in 1200 µL-20 °C pre-cooled 70% methanol aqueous internal standard extraction solution. The internal standard extraction solution was prepared by dissolving 1 mg of the standard (2-Chlorophenylalanine) in 1 mL of 70% methanol to make a 1000 µg/mL stock solution, which was further diluted with 70% methanol to prepare a 250 µg/mL internal standard solution. The mixture was vortexed (Vortex Mixer, VORTEX-5, Kyllin-Bell) every 30 minutes for 30 seconds each time, totaling 6 times. After centrifugation (Centrifuge, 5424R, Eppendorf) at 12,000 rpm for 3 minutes, the supernatant was collected, filtered through a microporous membrane (0.22 µm pore size) in preparation for UPLC-MS/MS analysis. The water used was ultrapure, while methanol, formic acid, and acetonitrile were all of chromatographic grade.

#### 2.2.2 Chromatogram and mass spectrometry acquisition conditions

Analysis of phenolic acid metabolites using Ultra-Performance Liquid Chromatography (UPLC, ExionLC™ AD, USA) Coupled with Tandem Mass Spectrometry (MS/MS).

Chromatographic conditions mainly included: (1) Column: Agilent SB-C18 1.8 µm, 2.1 mm x 100 mm; (2) Mobile Phase: phase A was ultrapure water with 0.1% formic acid, phase B was acetonitrile containing 0.1% formic acid; (3) Elution Gradient: At 0.00 min, mobile phase B was maintained at 5%; then linearly increased to 95% over 9.00 min and held at 95% for 1 min (9.00-10.00 min); subsequently, the phase B was rapidly reduced to 5% from 10.00 to 11.10 min, followed by a re-equilibration period at 5% until 14.00 min. (4) Flow Rate: 0.35 mL/min; Column Temperature: 40 °C; Injection Volume: 2 µL. The effluent was alternatively connected to an ESI-triple quadrupole-linear ion trap (QTRAP)-MS.

Mass Spectrometric Conditions mainly included: Electrospray ionization (ESI) source temperature was maintained at 500 °C with ion spray voltages (IS) set at 5500 V in positive ion mode and -4500 V in negative ion mode. Optimized ion source gas parameters included ion source gas I (GSI) at 50 psi, GSII at 60 psi, and air curtain (CUR) at 25 psi, with collision-induced ionization set to high. The triple quadrupole (QQQ) mass spectrometer was operated in multi-reaction monitoring (MRM) mode using nitrogen as collision gas at medium pressure. To ensure accurate and reliable results, the declustering potential (DP) and collision energy (CE) were systematically optimized to determine the optimal parameters for each MRM ion transition.

#### 2.2.3 Metabolic qualitative and quantitative analysis

Qualitative analysis of the compounds was performed based on secondary spectral information using the MWDB (MetWare Database). The original peak areas obtained from the detection were used as the relative content of phenolic acid metabolites in *F. suspensa* (**Supplementary Table S1**). Although the absolute content of the substances could not be quantified, the consistent detection conditions allowed for comparative analysis of the same compound across different samples.

### 2.3 Environmental variables

To test the correlations of different metabolites with environmental variables, we extracted 20 environmental variables (**Table 2**, **Supplementary Table S2**) of 10 F*. suspensa* populations under historical conditions (1970-2000) from the world climate database (https://www.worldclim.org/data/worldclim21.html), with 2.5 arcmin resolution.

**Table 2:** Content information of 104 environmental variables.

Abbreviation	Environmental variables	Unit
bio1	Annual Mean Temperature	°C
bio2	Mean Monthly Temperature Range	°C
bio3	Isothermality (MMTR/TAR)(*100)	–
bio4	Temperature Seasonality (standard deviation *100)	–
bio5	Max Temperature of Warmest Month	°C
bio6	Min Temperature of Coldest Month	°C
bio7	Temperature Annual Range	–
bio8	Mean Temperature of Wettest Quarter	°C
bio9	Mean Temperature of Driest Quarter	°C
bio10	Mean Temperature of Warmest Quarter	°C
bio11	Mean Temperature of Coldest Quarter	°C
bio12	Annual Precipitation	mm
bio13	Precipitation of Wettest Month	mm
bio14	Precipitation of Driest Month	mm
bio15	Precipitation Seasonality (Coefficient of Variation)	–
bio16	Precipitation of Wettest Quarter	mm
bio17	Precipitation of Driest Quarter	mm
bio18	Precipitation of Warmest Quarter	mm
bio19	Precipitation of Coldest Quarter	mm
elev	Elevation	m

### 2.4 Statistical analyses

We conducted metabolomic analysis using the Metware Cloud platform, which integrated multiple analytical modules: (1) “Violin Plots” were applied to assess inter-population distribution patterns of metabolites across 10 F*. suspensa* populations based on one-way analysis of variance (ANOVA); (2) “Advanced Significance Box Plot” were used to visualize differences between two groups using Student’s t-test; (3) “Cluster Heatmap” enabled population clustering based on metabolite profiles; (4) “Principal Component Analysis (PCA)” provided an overview of metabolic trends and overall variation in phenolic acids; (5) “Correlation Cluster Heatmap” were employed to evaluate relationships between metabolite levels and environmental variables using Pearson Correlation.

## 3 Results

### 3.1 Variation in phenolic acid metabolites among *F. suspensa* populations

**Figure 2:**
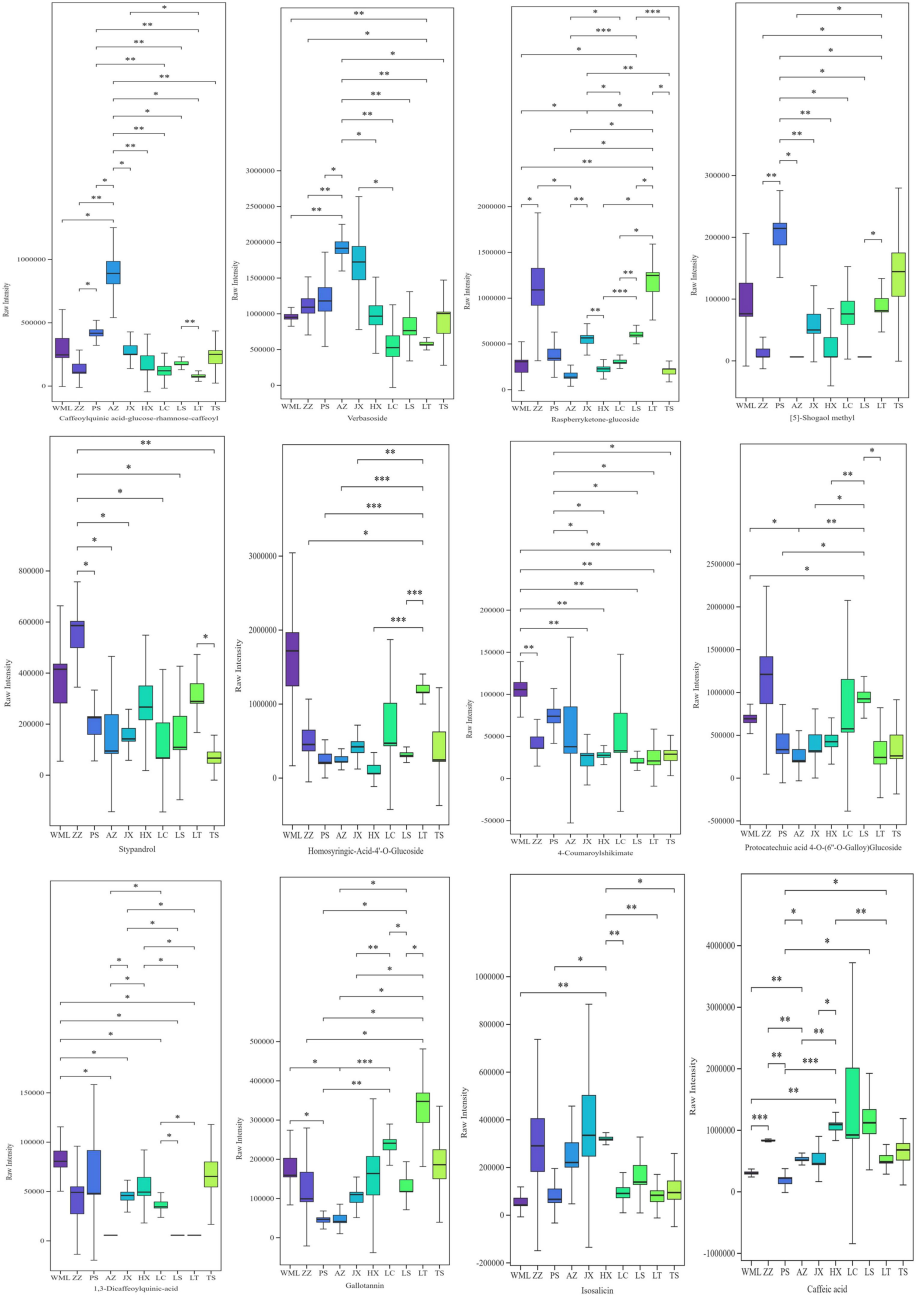
Advanced significance box plot of 12 phenolic acid metabolites in *F. suspensa*. Asterisks indicated significant differences according to student’s t-test (*p<0.05; **p<0.01; ***p<0.001).

All 30 phenolic acid metabolites we identified showed significant inter-population variation across 10 F*. suspensa* populations based on the one-way ANOVA (**Supplementary Figure S1**). Differences in metabolite levels between two groups were visualized using box plots based on Student’s t-test (**Figure 2**, **Supplementary Figure S2**). As shown in **Figure 2**, multiple metabolites displayed distinct population-specific accumulation patterns: Caffeoylquinic acid-glucose-rhamnose-caffeoyl was significantly higher in the AZ population than in the other nine populations. Verbasoside in AZ and Raspberryketone-glucoside in LT were significantly higher than in the other eight populations; [5]-Shogaol methyl in PS was significantly higher than in the other seven populations; Stypandrol in ZZ, Homosyringic-Acid-4’-O-Glucoside in LT, 4-Coumaroylshikimate in WML, and Protocatechuic acid 4-O-(6’’-O-Galloy)Glucoside in LS, were significantly higher than in the other six populations; 4-Coumaroylshikimate in PS, 1,3-Dicaffeoylquinic-acid in WML, Gallotannin in LT, and Isosalicin in HX were significantly higher than in other five populations.

**Figure 3:**
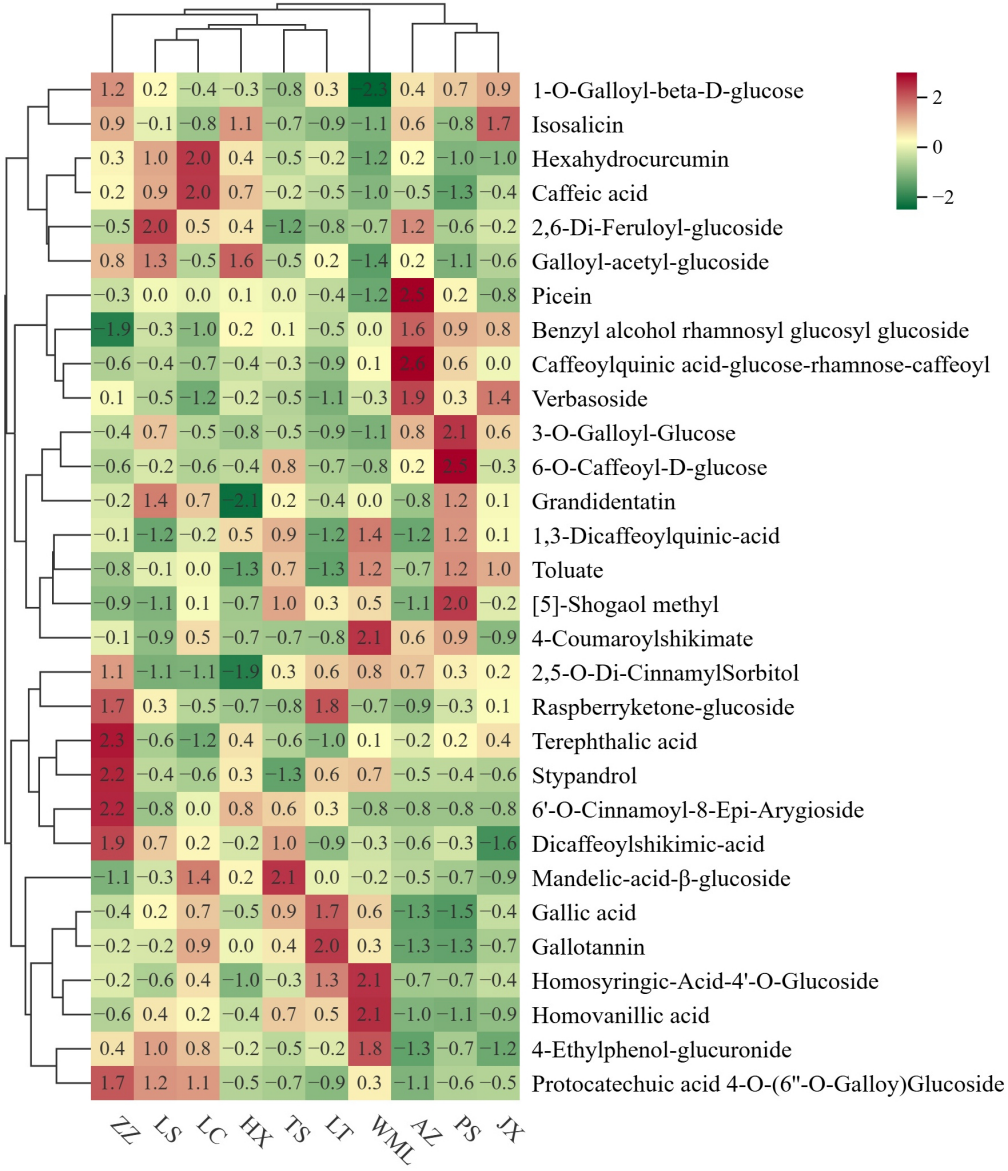
Cluster heatmap of phenolic acid metabolites in *F. suspensa*. Colour depth represents average intensity of metabolite contents. Red represents highest content, green represents lowest content.

**Figure 4:**
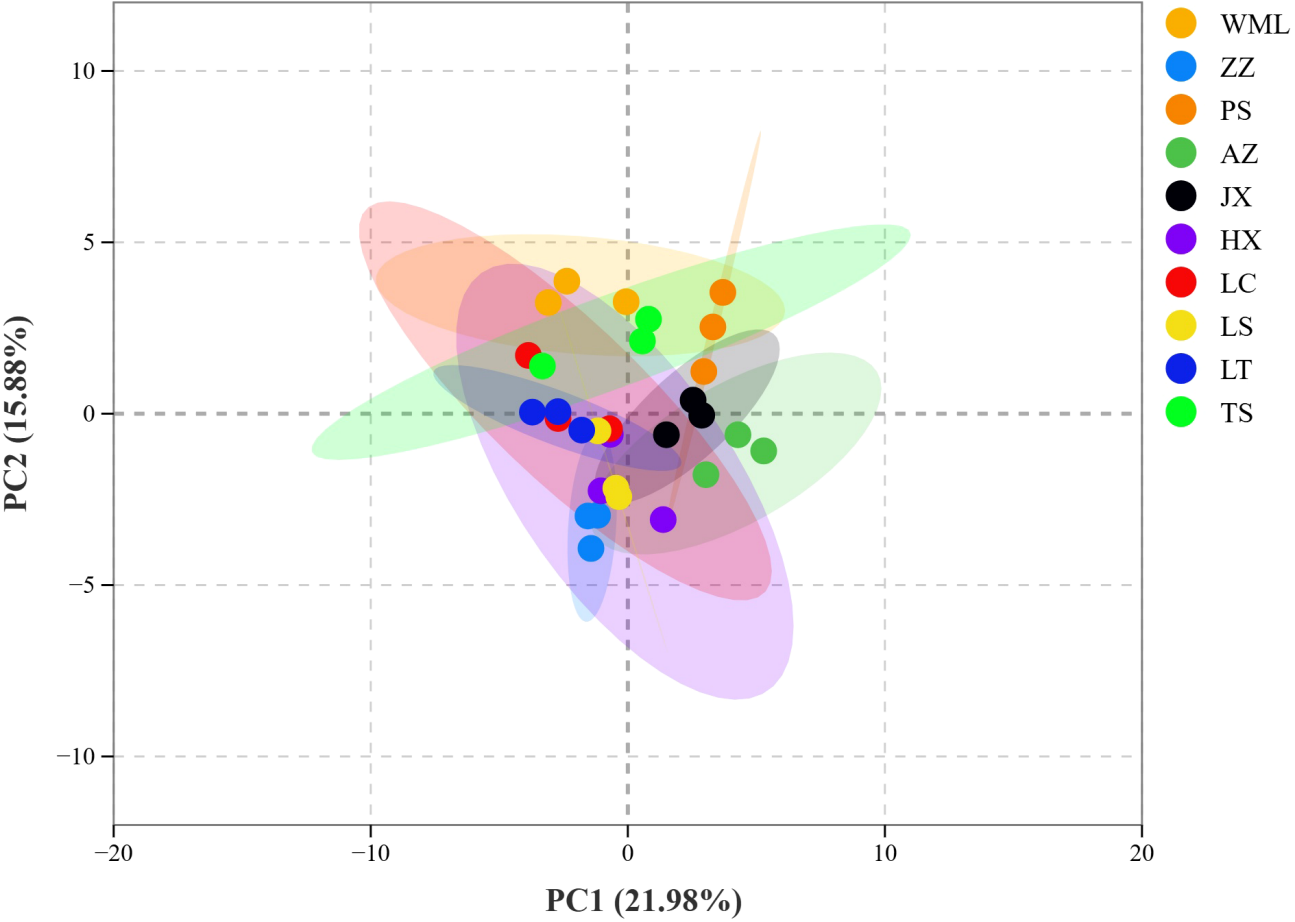
PCA score plot of phenolic acid metabolites in *F. suspensa*.

Having a comprehensive view of phenolic acids profile similarities and differences among *F. suspensa* populations, cluster heatmap (**Figure 3**) and PCA (**Figure 4**) were performed. Hierarchical clustering grouped the ten populations into five main clusters (**Figure 3**). Integrating these results with the significance testing in **Figure 2** revealed the following patterns: Cluster 1 (ZZ) showed high accumulation of seven metabolites (**Figure 3**), though only Stypandrol reached statistical significance (**Figure 2**); Cluster 2 (LS, LC, HX) displayed moderate levels of certain metabolites, with only Isosalicin being significantly enriched in HX; Cluster 3 (TS, LT) also displayed intermediate levels of certain metabolites, with Raspberryketone-glucoside, Homosyringic acid-4’-O-glucoside, and Gallotannin significantly enriched in LT; Cluster 4 (WML) accumulated six metabolites at high levels, among which 4-Coumaroylshikimate and 1,3-Dicaffeoylquinic acid were statistically significant; Cluster 5 (AZ, PS, JX) showed clear subclustering: Caffeoylquinic acid-glucose-rhamnose-caffeoyl and Verbasoside were significantly enriched in AZ, while [5]-Shogaol methyl and 4-Coumaroylshikimate were enriched in PS.

PCA further confirmed clear metabolic differentiation among *F. suspensa* populations (**Figure 4**). The first two principal components, Principal Component 1 (PC1, 21.98%) and 2 (PC2, 15.88%), collectively explained 37.86% of the total metabolic variance among samples. The score plot showed clear separation among all populations, reflecting substantial inter-population metabolic diversity. Population clustering within the PCA ordination demonstrated metabolic similarity: LS, HX, LC, and LT populations clustered closely, suggesting analogous phenolic acid profiles. Similarly, AZ, JX, and PS populations formed a distinct proximal group. Conversely, ZZ and WML populations occupied disparate positions within the ordination space, indicating pronounced metabolic divergence from each other. These patterns were consistent with the hierarchical clustering results (**Figure 3**), confirming robust population-level differentiation in phenolic acid composition across the studied *F. suspensa* populations.

### 3.2 Relationship between environmental variables and phenolic acid metabolites in *F. suspensa*

**Figure 5:**
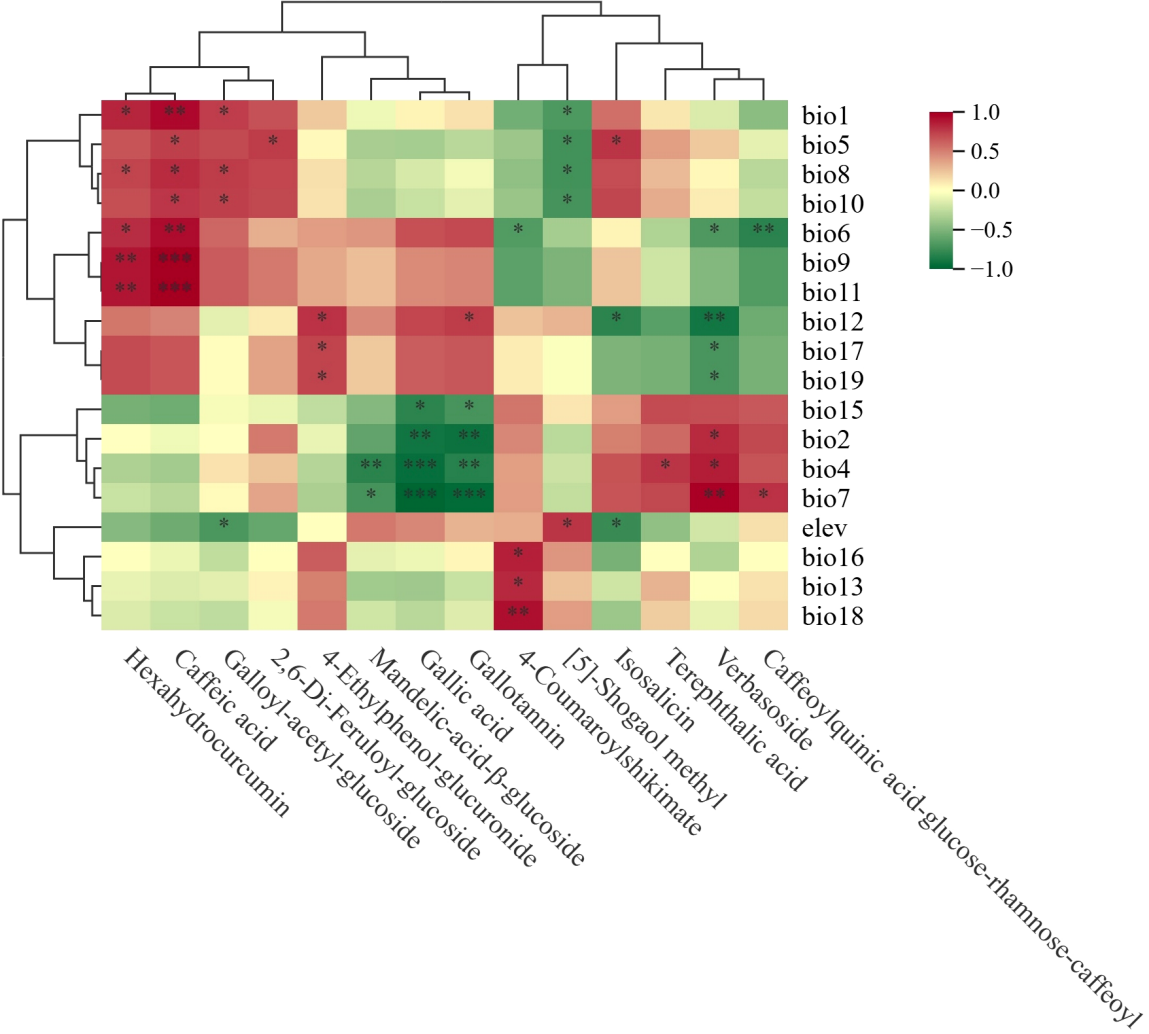
Cluster heatmap based on correlation between phenolic acid metabolites and the environmental variables. Red represents positive correlation, green represents negative correlation. “*” indicates statistical significant (p<0.05).

Correlation Cluster Heatmap was implemented to identify the major environmental variables governing the phenolic acid metabolites in *F. suspensa*. **Figure 5** presented significant correlations (*p*-values <0.05). We discovered that temperature significantly affected 13 phenolic acid metabolites, precipitation notably impacted 6 metabolites, and elevation affected 3 metabolites (**Figure 5**).

#### 3.2.1 Temperature-correlated metabolites

Eight phenolic acid metabolites exhibited significant positive correlations with temperature-related variables: Hexahydrocurcumin with bio1 (Annual Mean Temperature), bio8 (Mean Temperature of Wettest Quarter), bio6 (Min Temperature of Coldest Month), bio9 (Mean Temperature of Driest Quarter) and bio11 (Mean Temperature of Coldest Quarter); Caffeic acid with bio1, bio5 (Max Temperature of Warmest Month), bio8, bio10 (Mean Temperature of Warmest Quarter), bio6, bio9 and bio11; 2,6-Di-Feruloyl-glucoside with bio5; Terephthalic acid with bio4 (Temperature Seasonality); Galloyl-acetyl-glucoside with bio1, bio8, and bio10; Isosalicin with bio5; Verbasoside with bio2 (Mean Monthly Temperature Range), bio4, and bio7 (Temperature Annual Range); Caffeoylquinic acid-glucose-rhamnose-caffeoyl with bio7.

Seven metabolites exhibited significant negative correlations with temperature-related variables: Mandelic-acid-β-glucoside with bio4 and bio7; Gallic acid and Gallotannin were correlated with bio2, bio4, and bio7; 4-Coumaroylshikimate, Verbasoside and Caffeoylquinic acid-glucose-rhamnose-caffeoyl with bio6; [5]-Shogaol methyl with bio1, bio5, bio8, and bio10.

#### 3.2.2 Precipitation-correlated metabolites

Three metabolites showed significant positive correlation with precipitation-related variables: 4-Ethylphenol-glucuronide with bio12 (Annual Precipitation), bio17 (Precipitation of Driest Quarter), and bio19 (Precipitation of Coldest Quarter); Gallotannin with bio12; 4-Coumaroylshikimate with bio13 (Precipitation of Wettest Month), bio16 (Precipitation of Wettest Quarter), and bio18 (Precipitation of Warmest Quarter).

Four metabolites exhibited significant negative correlations with precipitation-related variables: Gallic acid and Gallotannin with bio15 (Precipitation Seasonality); Isosalicin with bio12; Verbasoside with bio12, bio17, bio19.

#### 3.2.3 Elevation-correlated metabolites

Elevation exhibited a significant negative correlation with Galloyl-acetyl-glucoside and Isosalicin, but a positive correlation with [5]-Shogaol methyl.

## 4 Discussion

The observed variation in phenolic acid metabolites among *F. suspensa* populations highlights the influence of environmental heterogeneity on its phytochemical profile. Given that *F. suspensa*, traditionally known as the “holy herb of the ulcer family”, holds significant value in Traditional Chinese Medicine for its pharmacological, ecological, and economic benefits (Commission, C. P, 2020), these findings provide important insights into how ecological factors shape the medicinal potential of this species.

In our study, several metabolites exhibited not only population-specific enrichment but also well-defined environmental correlations, underscoring their ecological and medicinal potential relevance. For instance, Verbascoside (also known as acteoside), a widely distributed phenylethanoid glycoside, exhibits broad pharmacological activities—including antioxidant, anti-inflammatory, antimicrobial, neuroprotective, and anticancer effects—supporting its therapeutic potential in wound healing, dermatitis, neurodegenerative disorders, and metabolic diseases (Das et al., 2016; Saha et al., 2024; Marčetić et al., 2025). In our study, verbascoside was significantly enriched in the AZ population and showed a positive correlation with temperature-related variables (bio2, bio4, bio7) but a negative correlation with precipitation (bio12, bio17, bio19) and Min Temperature of Coldest Month (bio6). Similarly, 4-Coumaroylshikimate, recognized for its antioxidant and anti-inflammatory functions (Lin et al., 2015; Barros et al., 2019), accumulated preferentially in WML and PS populations and was positively associated with wet-season precipitation (bio13, bio16, bio18) but negatively with Min Temperature of Coldest Month (bio6). Evidence supports the anti-inflammatory potential of Gallotannins and their relevance to chronic diseases, along with other bioactivities including antioxidant, antidiabetic, protein-precipitating, and antimicrobial effects (Kurniawan and Zahra, 2021; He, 2022). Our analysis revealed Gallotannin was significantly enriched in the LT population and correlated negatively with temperature variability (bio2, bio4, bio7) and Precipitation Seasonality (bio15), but positively with Annual Precipitation (bio12). Isosalicin, a salicylic acid precursor with anti-inflammatory and analgesic properties (Havén and Julkunen-Tiitto, 2003; Cheng et al., 2025), accumulated in HX and responded positively to Max Temperature of Warmest Monthhigh (bio5) but negatively to Annual Precipitation (bio12) and elevation. Hexahydrocurcumin, known for antioxidant and anti-inflammatory activities (Li et al., 2012; Srimuangwong et al., 2012), showed significant positive correlations with temperature variables (bio1, bio6, bio8, bio9, bio11). Caffeic acid, a ubiquitous phenolic acid with a catechol moiety, confers strong antioxidant capacity and diverse pharmacological properties such as anti-inflammatory, antimicrobial, and anticancer activities. It has potential applications in managing diabetes and neurodegenerative diseases (Zhao et al., 2022; Yazar et al., 2025). Recent study indicated its enrichment in high-altitude ecotypes (Qian et al., 2025). However, our study revealed that Caffeic acid exhibit no significant correlation with altitude, but rather significant positive correlations with multiple temperature variables (bio1, bio5, bio6, bio8, bio9, bio10, bio11), suggesting a complex biosynthetic regulatory mechanism in plants. These metabolite-specific environmental responses together illustrated how environmental heterogeneity regulatefs the phenolic acid metabolites of *F. suspensa* and thus affects its medicinal potential.

Low temperature represents a primary constraint on *F. suspensa* cultivation, limiting suitable geographic areas for its planting and overall yield (Cui et al., 2024). Recent research on cold resistance indicates that *F. suspensa* employs adaptive strategies, including increased phenolic acid accumulation to enhance osmotic potential and mitigate cold damage (Cui et al., 2025). This aligns with evidence of cold adaptive differentiation among natural populations (Li et al., 2020), differential expression in cold-tolerance pathways (Li et al., 2021), and genetic divergence driven by Pleistocene climate fluctuations (Fu et al., 2014). Mantel tests and redundancy analysis further link population genetic differentiation to geographical distance, temperature, and latitude (Fu et al., 2016), consistent with our finding that temperature governs phenolic acid-based adaptation.

Our correlation analyses robustly established temperature as the principal abiotic factor sculpting phenolic acid profiles. Eight metabolites (Hexahydrocurcumin, Caffeic acid, 2,6-Di-Feruloyl-glucoside, Terephthalic acid, Galloyl-acetyl-glucoside, Isosalicin, Verbasoside, and Caffeoylquinic acid-glucose-rhamnose-caffeoyl) showed strong positive correlations with temperature, while seven metabolites (Mandelic-acid-β-glucoside, [5]-Shogaol methyl, Gallic acid, Gallotannin, 4-Coumaroylshikimate, Verbasoside, Caffeoylquinic acid-glucose-rhamnose-caffeoyl, and [5]-Shogaol methyl) exhibited negative correlations, suggesting complex biosynthetic regulation. Precipitation played a secondary role, significantly influencing six metabolites in our study: positive correlations with 4-Ethylphenol-glucuronide, Gallotannin and 4-Coumaroylshikimate, and negative correlations with Isosalicin, Verbasoside, Gallic acid and Gallotannin. This aligns with meta-analyses indicating that decreased precipitation generally promotes phenolic levels (Sun et al., 2023), supporting our observation of decreased Isosalicin, Verbasoside, Gallic acid and Gallotannin as potential dry-adaptation responses.

Population genomic studies corroborate these environmental links, identifying candidate genes for local adaptation associated with solar radiation, temperature, and water availability (Li et al., 2020), and revealing genetically distinct groups shaped by divergent selection driven by multiple environmental variables (Yang et al., 2017). Our results extend this understanding by delineating specific correlations between precipitation, temperature, and individual metabolite abundances *F. suspensa*.

Collectively, our findings demonstrate significant inter-population variation in phenolic acid metabolites of *F. suspensa*, is primarily driven by temperature heterogeneity, with precipitation acting as a secondary factor and elevation playing only a minimal role. By decoding key environment-metabolite relationships, we provide a framework for climate-smart cultivation, germplasm conservation, and metabolite-directed breeding. However, the complexity of secondary metabolism underscores the need for future multi-factorial studies that integrate metabolomic and transcriptomic approaches to clarify how environmental variables such as temperature and precipitation regulate rate-limiting steps in phenolic acid biosynthesis. We also recognize certain limitations in this study, including restricted population sampling, the lack of soil physicochemical analysis, and insufficient seasonal or molecular-level data—all of which may influence the interpretation of metabolite variation. Given that soil properties such as pH, nutrient availability, and organic matter content interact strongly with climatic factors in shaping secondary metabolism, future work should integrate soil analysis with multi-omics approaches to more comprehensively elucidate how environmental variables regulate critical steps in phenolic acid biosynthesis.

## 5 Conclusion

This study revealed substantial variation in the content of 30 phenolic acid metabolites across 10 F*. suspensa* populations. Temperature emerged as the dominant environmental modulator, significantly influencing the accumulation of over two-thirds of phenolic acids, while precipitation played a secondary role and elevation exhibited only minimal effects. These findings offer important insights into the ecological adaptation of *F. suspensa* and provide a scientific basis for future metabolite-oriented breeding and cultivation strategies.

## Data Availability

The original contributions presented in the study are included in the article/**Supplementary Material**. Further inquiries can be directed to the corresponding author/s.
