# Supplementary material for: Variations in phenolic acid metabolites among Forsythia suspensa populations in response to environmental heterogeneity
Source: Front Plant Sci. 2025 Nov 5;16:1683181. doi: 10.3389/fpls.2025.1683181 (PMC12626952; doi:10.3389/fpls.2025.1683181)
Supplement: Supplementary file 2 [file Image1.pdf]

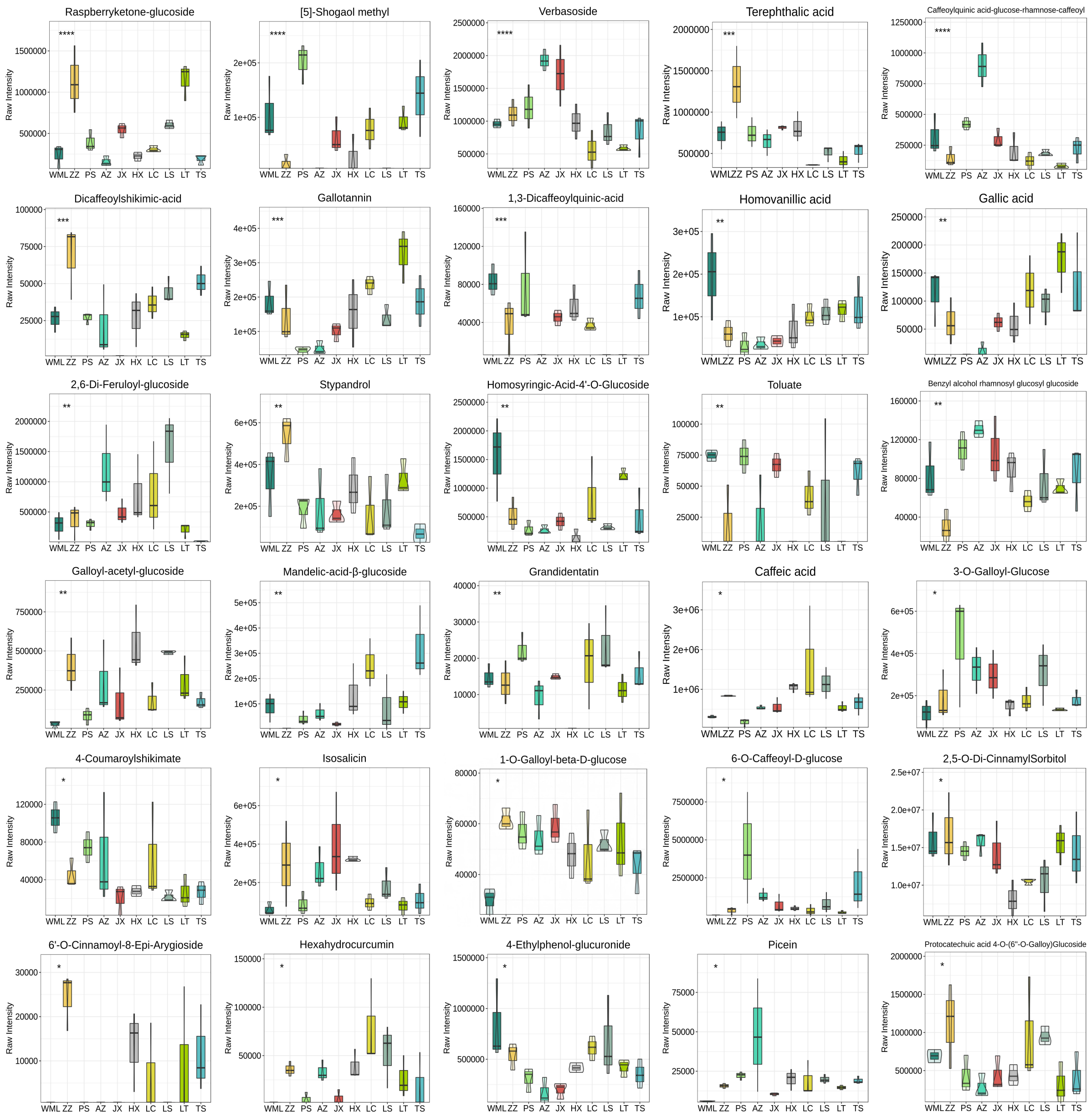

Figure S1 Violin Plot of 30 phenolic acid metabolites in *F. suspensa*.

Asterisks indicated significant differences according to ANOVA (*, p<0.05; **, P < 0.01; ***, p<0.001; ****, P < 0.0001).
