# Supplementary material for: Variations in phenolic acid metabolites among Forsythia suspensa populations in response to environmental heterogeneity
Source: Front Plant Sci. 2025 Nov 5;16:1683181. doi: 10.3389/fpls.2025.1683181 (PMC12626952; doi:10.3389/fpls.2025.1683181)
Supplement: Supplementary file 3 [file Image2.pdf]

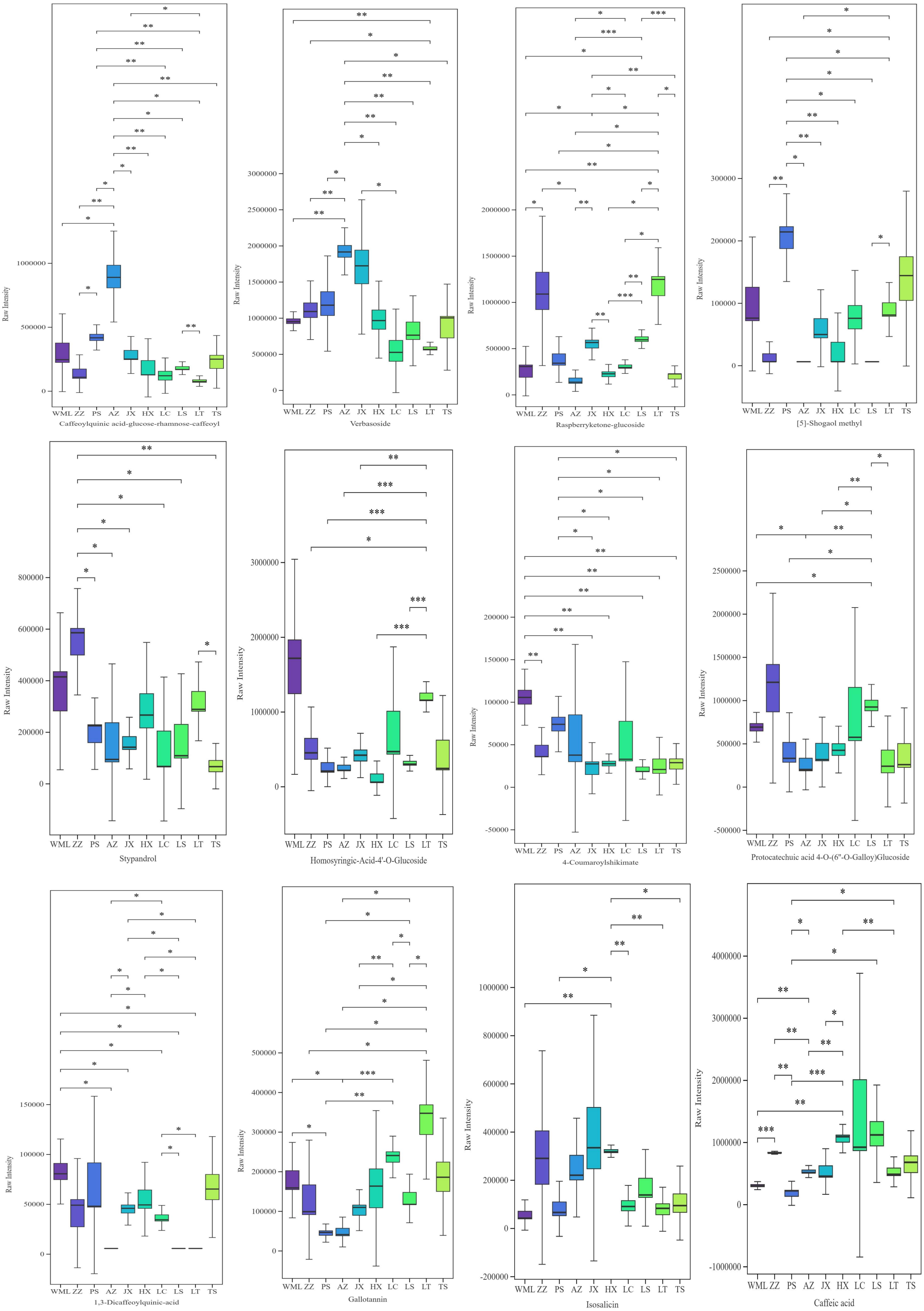
Raw Intensity
WML ZZ PS AZ JX HX LC LS LT TS
Caffeoylquinic acid-glucose-rhamnose-caffeoyl
Verbasoside
Raspberryketone-glucoside
[5]-Shogaol methyl
Stypandrol
Homosyringic-Acid-4'-O-Glucoside
4-Coumaroylshikimate
Protocatechuic acid 4-O-(6''-O-Galloy)Glucoside
1,3-Dicaffeoylquinic-acid
Gallotannin
Isosalicin
Caffeic acid

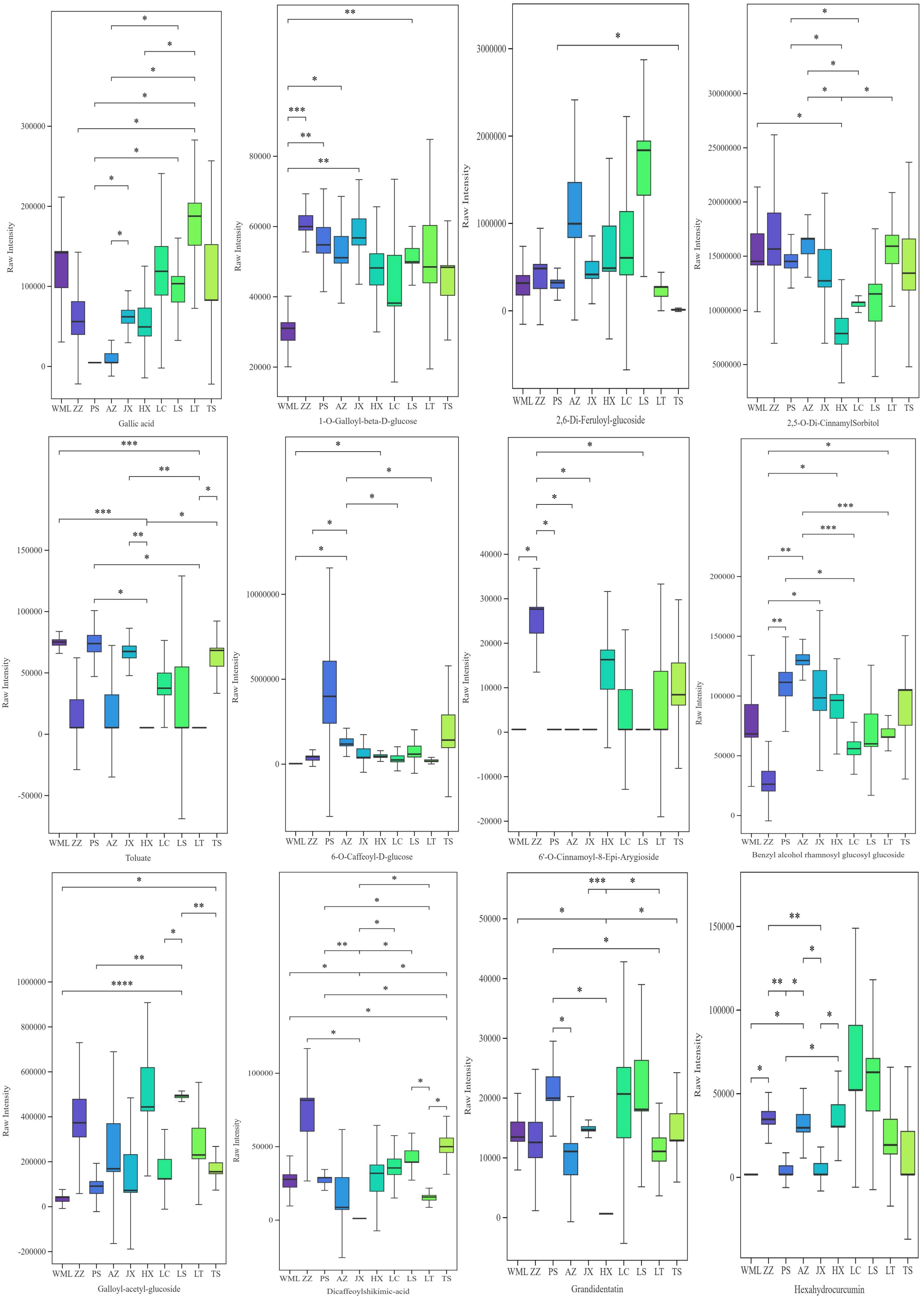

Raw Intensity
WML ZZ PS AZ JX HX LC LS LT TS
Gallic acid
1-O-Galloyl-beta-D-glucose
2,6-Di-Feruloyl-glucoside
2,5-O-Di-CinnamylSorbitol
Toluate
6-O-Caffeoyl-D-glucose
6'-O-Cinnamoyl-8-Epi-Arygioside
Benzyl alcohol rhamnosyl glucosyl glucoside
Galloyl-acetyl-glucoside
Dicaffeoylshikimic-acid
Grandidentatin
Hexahydrocurcumin

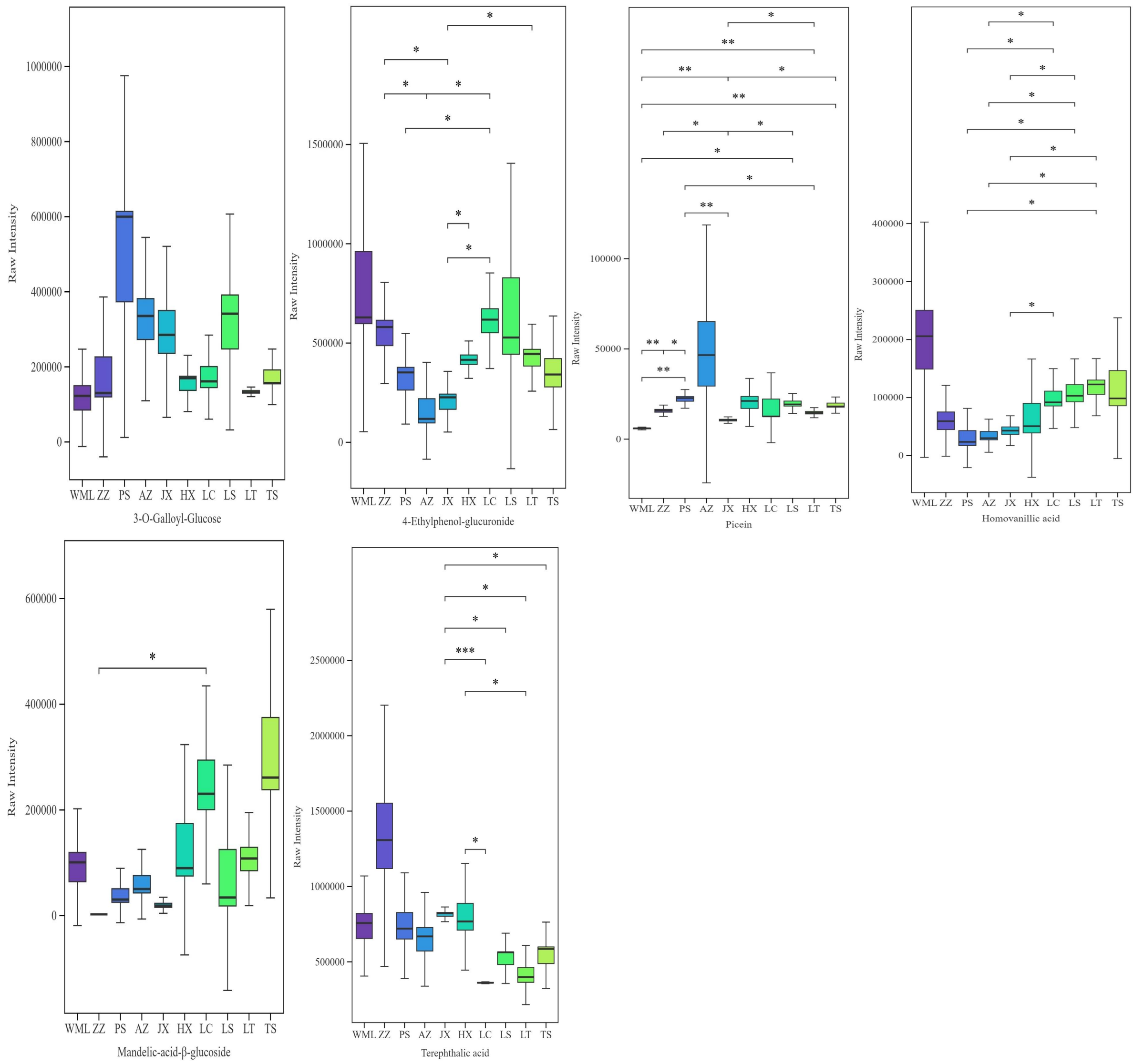

Figure S2 Advanced Significance Box Plot of 30 phenolic acid metabolites in *F. suspensa*. Asterisks indicated significant differences according to Student's t-test (*, p<0.05; **, P < 0.01; ***, p<0.001).
